# High concentration factor diffractive microlenses integrated with CMOS single-photon avalanche diode detector arrays for fill-factor improvement

**DOI:** 10.1364/AO.388993

**Published:** 2020-05-08

**Authors:** Peter W. R. Connolly, Ximing Ren, Aongus McCarthy, Hanning Mai, Federica Villa, Andrew J. Waddie, Mohammad R. Taghizadeh, Alberto Tosi, Franco Zappa, Robert K. Henderson, Gerald S. Buller

**Affiliations:** 1 Institute of Photonics and Quantum Sciences, School of Engineering and Physical Sciences, Heriot-Watt University, Edinburgh, EH14 4AS, UK; 2 Dipartimento di Elettronica, Informazione e Bioingegneria, Politecnico di Milano, Milano 20133, Italy; 3 School of Engineering, Institute for Integrated Micro and Nano Systems, The University of Edinburgh, Edinburgh, EH9 3JL, UK; 4 Current address: Micron School of Materials Science and Engineering, Boise State University, Boise, Idaho 83725, USA

## Abstract

Large-format single-photon avalanche diode (SPAD) arrays often suffer from low fill-factors—the ratio of the active area to the overall pixel area. The detection efficiency of these detector arrays can be vastly increased with the integration of microlens arrays designed to concentrate incident light onto the active areas and may be refractive or diffractive in nature. The ability of diffractive optical elements (DOEs) to efficiently cover a square or rectangular pixel, combined with their capability of working as fast lenses (i.e., ∼f/3) makes them versatile and practical lens designs for use in sparse photon applications using microscale, large-format detector arrays. Binary-mask-based photolithography was employed to fabricate fast diffractive microlenses for two designs of 32×32 SPAD detector arrays, each design having a different pixel pitch and fill-factor. A spectral characterization of the lenses is performed, as well as analysis of performance under different illumination conditions from wide- to narrow-angle illumination (i.e., f/2 to f/22 optics). The performance of the microlenses presented exceeds previous designs in terms of both concentration factor (i.e., increase in light collection capability) and lens speed. Concentration factors greater than 33× are achieved for focal lengths in the substrate material as short as 190µm, representing a microlens f-number of 3.8 and providing a focal spot diameter of <4µm. These results were achieved while retaining an extremely high degree of performance uniformity across the 1024 devices in each case, which demonstrates the significant benefits to be gained by the implementation of DOEs as part of an integrated detector system using SPAD arrays with very small active areas.

## 1. INTRODUCTION

The use of complementary metal–oxide–semiconductor (CMOS) fabrication processes has enabled the manufacture and production of large-format, monolithically integrated single-photon imaging arrays. Two-dimensional single-photon avalanche diode (SPAD)-based arrays based on deep-submicrometer CMOS technologies are becoming more commonplace [1–5], with such devices utilizing integrated in-pixel circuitry to perform quenching, counting, and/or timing functionalities. These CMOS SPAD detector arrays have been used effectively in a number of applications, for example, depth profiling at short [6] and long ranges [7] and time-resolved fluorescence lifetime imaging (FLIM) [8]. One of the biggest drawbacks of this design is that the circuitry requires space on the pixel, which could otherwise be dedicated to photodetection, thereby reducing the effective single-photon detection efficiency (SPDE) of the device. The *fill-factor* is the ratio of the pixel’s photosensitive area to the total area of the pixel, and it is often less than 5%, although can be as high as 60% in SPAD arrays with reduced functionality (e.g., arrays without in-pixel timing electronics) [9].

Increasing the available effective fill-factor has been attempted through the use of 3D stacking the electronics behind the active (i.e., photosensitive) area [10,11] and the use of microlens arrays, both refractive [12,13] and diffractive [14,15] in nature, to varying degrees of success. The manufacture of microlenses requires the use of a variety of micro- and nanofabrication techniques to create the 2D arrays of microscale optical lens elements necessary to focus incident light onto the active areas of each SPAD in an array. Refractive-type microlenses may be fabricated using ink-jet [16] or injection molding [17] techniques, but they are most commonly produced using the *resist-reflow* method [18–21], whereby columns of photoresist are melted such that surface tension produces the required spherical microlens form. This form is then etched into the substrate, generally using reactive ion etching (RIE). For a square pixel, the maximum fill-factor achievable by refractive lenses with a circular base, as attained with the reflow technique, is 78.5%. Diffractive microlenses rely on a blazed grating structure containing the surface profile of the equivalent refractive lens, divided into slices of ∼2π width. This surface structure is typically obtained via multistage photolithography using binary masks and either RIE or wet etching [14,15,22]. Electron-beam lithography can be employed in a similar fashion [23], allowing higher-resolution features but requiring much longer write times. Direct laser writing may also be used [24], a method which enables a smooth progression in height across an etched structure by imposing a gradient to the laser power during exposure as opposed to the stepped structure produced by binary-mask-based photolithography. This has the benefit of producing elements of higher diffraction efficiency in only a single stage of lithography, though it requires much more advanced and expensive technology to produce. Diffractive optical elements (DOEs) have a stronger wavelength dependence than their refractive counterparts [25]; however, the possibility to retain circular symmetry within a rectangular bound allows DOEs to achieve up to 100% coverage. This larger achievable fill-factor provides a much greater benefit, especially in sparse photon applications, than would be possible using a reflow-type refractive component. The ongoing development of CMOS-based SPAD technologies has led to wafer-level micro-optics becoming more common [26–29]; however, these processes are incompatible with non-CMOS-based devices. The development of short-wave infrared SPADs, such as InGaAs/InP arrays [30,31] and Ge-on-Si detectors [32,33], justifies the further developments of stand-alone micro-optics, which can be integrated post fabrication.

This work presents diffractive microlens arrays designed for two different 32×32 Si CMOS SPAD arrays in order to improve their effective fill-factors, and therefore SPDE, for use in sparse photon environments. One of the SPAD detector arrays used in this study (MF32) had detector elements with an active area of only 7µm in diameter (and a corresponding fill-factor of approximately 1.5%). As the diffraction-limited focal spot size achievable by a lens scales with focal distance, fast lenses were required to ensure as much light falls within this region as possible. The lens arrays were fabricated on fused silica substrates, which were then flip-chip bonded onto the sensors. The performance of the lenses was characterized in terms of focal spot, concentrating power, and spatial uniformity over a large spectral range (λ=500−800nm) and illumination f-numbers from f/2 to f/22.

## 2. DIFFRACTIVE MICROLENS DESIGN AND FABRICATION

**Fig. 1.**
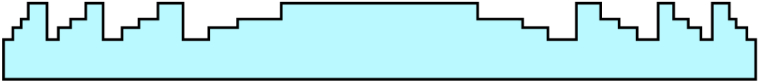
Step profile of an etched DOE of phase levels L=4.

Binary-mask-based photolithography was used in the fabrication of the microlens arrays produced in this work. In order to achieve maximum diffraction efficiency of a DOE at a given wavelength λ, the profile of the equivalent refractive lens is sliced into widths equal to 2π to achieve phase matching across the element. The elemental thickness t required to achieve the 2π phase difference is given by Eq. (1):

(1) $$t=\frac{\lambda }{n_{g}-1},$$

where $n_{g}$ is the refractive index of the lens medium [34]. To ensure the diffracted light in such a device is contained predominantly within a single order, a blazed grating structure is required (i.e., no surface curvature) [35]. The smoother the gradient of the surface, the higher the efficiency of the lens; however, the fabrication process used to produce the lenses in this paper required the depth to be etched in steps, resulting in the staircase-like profile shown in Fig. 1. The focusing efficiency $\eta_{d}$ of a stepped DOE may be estimated from the first-order diffraction theory and related to the number of phase levels used to etch the lens L as [36]

(2) $$\eta_{d}={\left|\frac{\sin(\pi /L)}{\pi /L}\right|}^{2}.$$

**Fig. 2.**
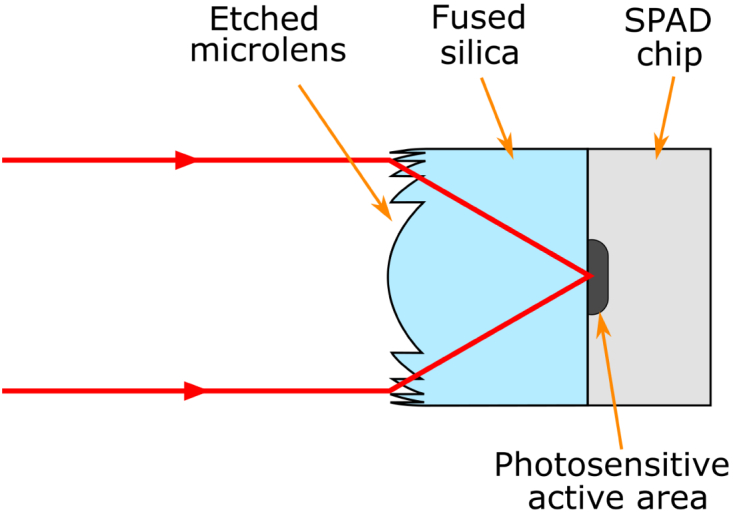
Schematic of infinite conjugate microlens configuration designed to image an object placed at infinity onto the active area of a SPAD pixel. The design focal length is equal to the thickness of the fused silica substrate divided by its refractive index.

As Eq. (2) indicates, the more phase levels used (L), the higher the focusing efficiency of the DOE. In this work, binary-mask-based photolithography is employed, with each mask allowing a factor of 2 increase in the number of levels etched (i.e., $L=2^{n_{m}}$, where $n_{m}$ is the number of masks). Reactive ion etching (RIE) using photolithography has been shown to ensure vertical sidewalls are maintained while the aspect ratio (i.e., the etch depth divided by the feature width) remains less than ∼1.2 [37]. For the diffractive lenses discussed in this paper, this was achieved by reducing the number of phase levels towards the outer edges of each lens where the aspect ratio would otherwise rise above this limit. This occurs, for the design wavelengths presented in this paper, when the lateral feature size required drops below ∼1.2−1.5µm. Taking these limitations into account, four masks were designed for both cases presented here, allowing for a maximum of $2^{4}=16$ levels. Both designs were also such that the lenses operated in an infinite conjugate configuration, i.e., were designed to focus a collimated light source or image an object placed at infinity from the system as indicated in Fig. 2.

**Fig. 3.**
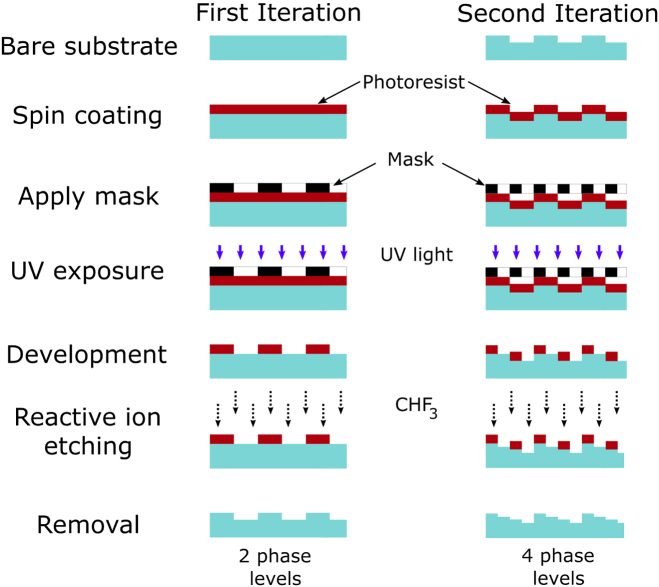
Binary-mask-based photolithography DOE fabrication process.

**Table 1.** Microlens Design Parameters

Design Parameter	MiSPIA	MF32
Array format	32×32	
Pixel pitch	150µm	50µm
Substrate dimensions	6mm×6mm	3.5mm×2.6mm
Design wavelength	808 nm	580 nm
Focus distance (substrate thickness)	620µm	190µm
Lens configuration	Object at Infinity	
Maximum phase levels	14	12
Pixel active area	ϕ=30µm	ϕ=7µm
Theoretical spot diameter [from Eq. (3)]	5.6µm	3.7µm

The resulting first-order focal spot is diffraction limited in size, the diameter of which may be calculated according to the first minima in the Airy disc pattern formed by its Bessel function [38], which, for plane wave illumination, is given by

(3) $$d=2.44\frac{f\lambda }{n_{g}D},$$

where λ is the wavelength of incident light, D is the aperture diameter, f is the focal length at that wavelength in the substrate, and $n_{g}$ is the refractive index of the substrate. The fabrication procedure is shown schematically in Fig. 3.

## 3. MICROLENS DESIGN SPECIFICATIONS

Microlens arrays were designed and fabricated for two types of 32×32 Si CMOS SPAD detector arrays, each with different design specifications for pixel pitch, active area size, and fill-factor. To ensure diffractive optics are useful in a wide range of applications, it can be advantageous to use as thin a substrate as possible. The lenses designed for the work presented in this paper build on that done in Ref. [15], first reducing the focal length from 1 mm to 620µm for lenses designed for the same image sensor. Second, a design is made for a sensor of smaller dimensions and with a much smaller active area, with an even shorter focal length of only 190µm.

### A. MiSPIA Image Sensor

The first chip integrated with a microlens array was a Si CMOS SPAD array called the microelectronic single-photon 3D imaging array for low-light high-speed safety and security applications (MiSPIA), the result of a European Commission funded project [39]. The MiSPIA sensor, effective within the wavelength range 300–900 nm, contains a 32×32 array of square pixels of 150µm pitch, each with a 30µm diameter circular active area equating to a fill-factor of 3.14% [5]. The microlens array was designed to match exactly the dimensions of the detector array, and it has a design wavelength of λ=808nm, etched into a substrate of thickness 620µm. The number of phase levels necessarily reduces from 14 in the center down to 2 in the corner regions of each lens, as the feature sizes in these areas would otherwise be smaller than 1.5µm, which becomes difficult to resolve by the photoresist during development. The refractive index of fused silica at the design wavelength of λ=808nm is 1.453 [40]. From Eq. (3), the diffraction-limited spot size is thus 5.6µm in diameter, which compares as just under 20% the size of the 30µm diameter detector active area. The design parameters for the MiSPIA microlenses are summarized in Table 1.

**Fig. 4.**
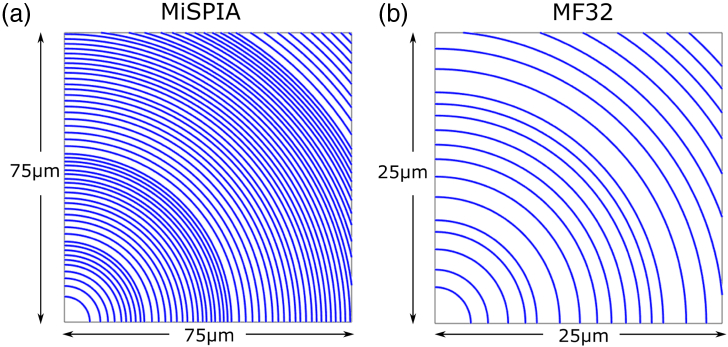
Model of etch zone radii for 90° segment (i.e., quarter) of a single lens in each array: (a) MiSPIA; (b) MF32.

**Fig. 5.**
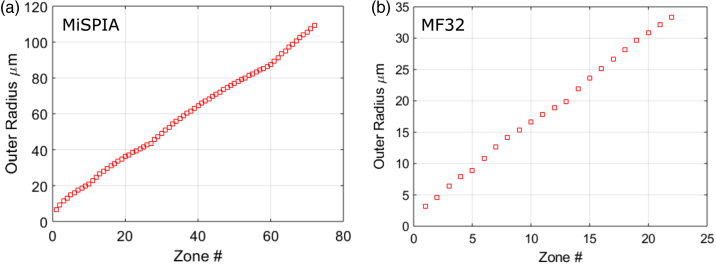
Outer radii of every zone in each microlens array: (a) MiSPIA; (b) MF32.

**Fig. 6.**
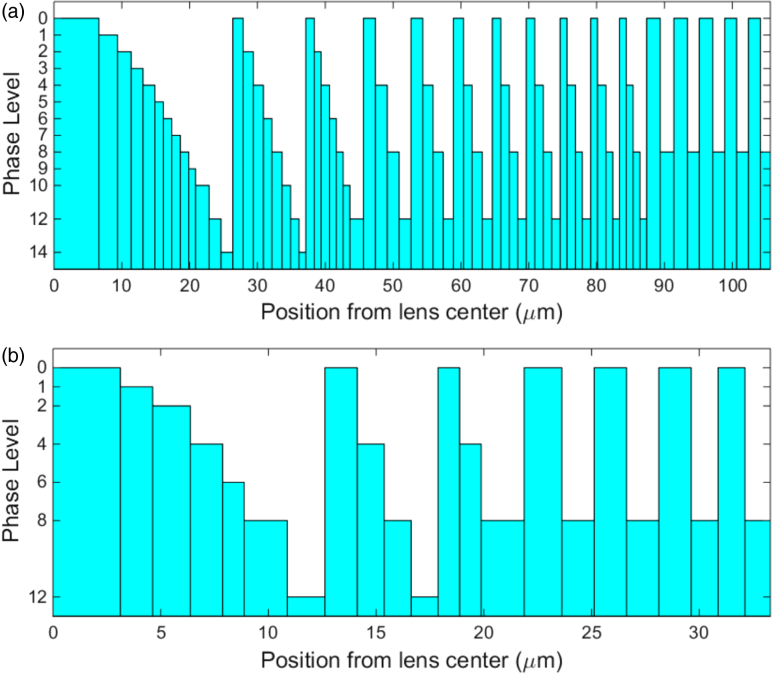
Etch zone phase level profile for each microlens array: (a) MiSPIA; (b) MF32. The etch depths for each phase level l are calculated as $l\times \frac{t}{16}$, where t is the total elemental thickness derived using Eq. (1).

### B. MF32 Image Sensor

Similarly to the MiSPIA chip, the MF32 chip is a 32×32 Si CMOS SPAD array effective within the wavelength range 300–900 nm; however, the pitch is significantly smaller at 50µm, and an active area of 7µm in diameter means a fill-factor of ∼1.5% [41]. The MF32 SPAD array is a result of the *MEGAFRAME* project [42], the purpose of which was to develop a large-format single-photon imaging arrays in a low-cost, deep-submicrometer CMOS platform, with 32×32 and 128×128 format devices manufactured. The microlens array must again match the dimensions of the SPAD array onto which it is being bonded. The design wavelength in this case was 580 nm, and to achieve a focal spot small enough to fall comfortably within the active area, a substrate of 190µm thickness is used. The refractive index of fused silica at a wavelength of λ=580nm is 1.459 [40]. From Eq. (3), the diffraction-limited spot size is thus 3.7µm in diameter. This is equivalent to >50% of the diameter of the active area onto which it must be focused, resulting in a much smaller margin for error with both alignment and fabrication than for the MiSPIA microlens array. Fewer phase levels are possible (a maximum of 12 rather than 14) due to the much smaller dimensions of the MF32. The design parameters of the microlenses for both sensors are provided for comparison in Table 1.

**Fig. 7.**
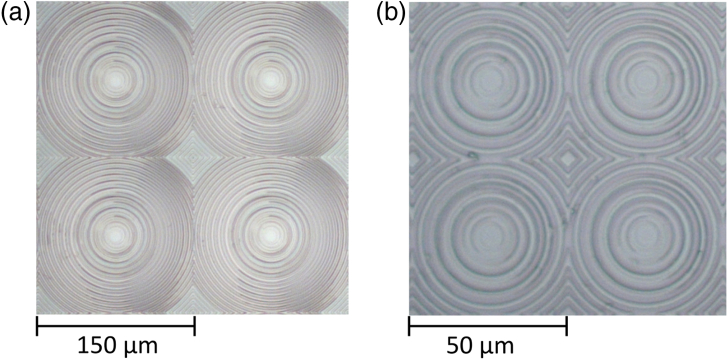
Microscope images showing 2×2 lenses from a section of each microlens array: (a) MiSPIA; (b) MF32.

The lens etch zones were modeled for each array, with the etch radii for quarter segments of a single pixel shown in Figs. 4 and 5, while Fig. 6 provides the phase level profiles of each. Figure 7 shows micrographs of the resulting lens arrays once fabricated.

**Fig. 8.**
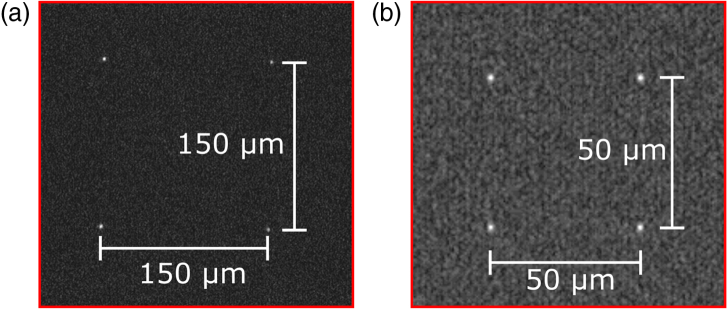
Focal spots of four lenses on (a) MiSPIA array under λ=808nm illumination; (b) MF32 array under λ=580nm illumination. The spot size diameters were measured as 4.8±0.9µm and 3.3±0.2µm, respectively.

**Fig. 9.**
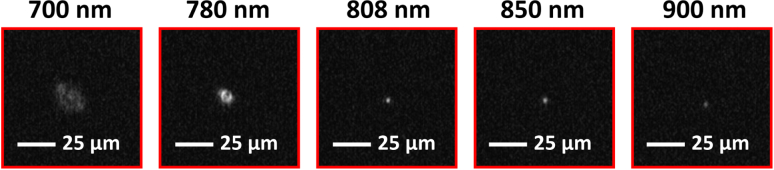
Spot profile obtained at the back surface of the substrate from an individual lens in the MiSPIA array when illuminated by 700, 780, 808, 850, and 900 nm wavelength radiation.

## 4. INTEGRATION AND SYSTEM CHARACTERIZATION

In order to ensure that only high-quality microlens arrays were used, a preliminary inspection was conducted prior to integration with the SPAD array. The focal plane of the microlens array was inspected by imaging the back surface of the microlens substrate onto a charge-coupled device (CCD) camera. By illumination with a collimated laser, the focused spots could be magnified and imaged onto the CCD using a microscope objective. The resulting spots were imaged and the intensity profile extracted, from which their sizes were calculated. Due to the error in ascertaining the Airy minima, we characterized the spot diameter as the full width at $1/e^{2}$ of the peak value. From the images, shown in Fig. 8, the spot diameters were determined as 4.8±0.9µm for the MiSPIA lens and 3.3±0.2µm for the MF32 lens. The spot sizes may appear slightly smaller than the previously quoted diffraction-limited size (5.6µm and 3.7µm for MiSPIA and MF32 lenses, respectively). This is mainly due to the $1/e^{2}$ diameter measurements always being less than the theoretical Airy minimum described in Eq. (3).

Figure 9 shows how the spot size of these diffractive microlenses varies when illuminated with individual discrete wavelengths in the range 700  to 900 nm. The images were taken at the same image plane (i.e., the back surface of the microlens substrate) using an individual lens from the MiSPIA microlens array.

**Fig. 10.**
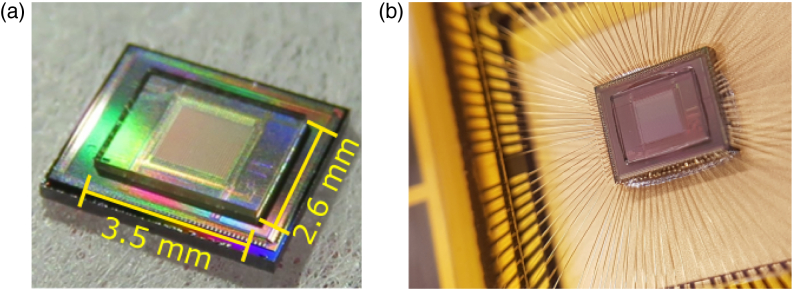
Close-up images of integrated microlens array and MF32 SPAD detector array using a macro-lens on a CCD camera: (a) post-integration; (b) after wire bonding.

An ACCµRA100 flip-chip bonder was used to align and bond the microlens arrays with the chips using a UV curing optical adhesive. In the case of the MiSPIA chip, fiducial markings on the detector array and microlens arrays were used for reference points during alignment, and a force of 0.2 kg was used for bonding. This recipe provided a high accuracy of alignment while not applying enough pressure to damage the chip, and it used the same parameters as those used in Ref. [15]. The MF32 bonding uses a similar process; however, the absence of fiducial markings meant alignment was conducted using the lens and detector active area centers. The dimensions of the MF32 microlens array are also much smaller than those of the MiSPIA array (3.5mm×2.6mm, compared to the MiSPIA microlens array dimensions of 6mm×6mm), meaning an identical force results in an increase in pressure of ∼×4. To prevent damaging the detector chip and/or the microlens substrate, the applied force was reduced to 40 g, which was found not to affect alignment accuracy. Figure 10 shows a microlens array and MF32 chip immediately post-integration and after subsequent wire bonding.

**Fig. 11.**
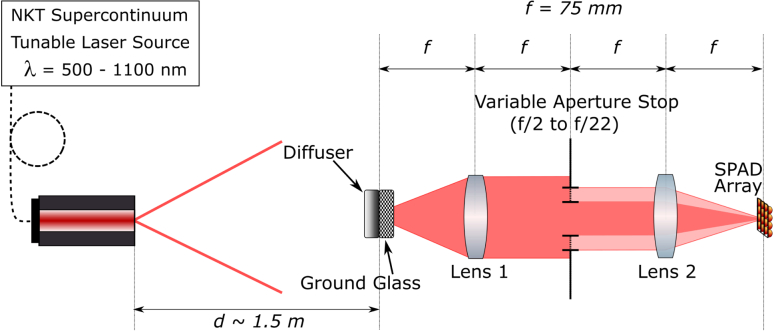
Optical setup used to characterize the concentration factors and uniformities of the microlens-enhanced SPAD arrays. The variable aperture stop allows measurements at different illumination f-numbers. The NKT supercontinuum laser system, using an acousto-optical tunable filter (AOTF), can output discrete wavelengths from 500  to 1100 nm.

In order to characterize the performance of the microlens arrays, a double telecentric 4f system was employed [15] as illustrated in Fig. 11. The key benefits of such a system include providing a uniform illumination at the image plane, a constant magnification independent of object position, and no off-axis distortion.

A tunable illumination was achieved using an NKT supercontinuum laser providing emission between 500  and 1100 nm. A divergent beam emitted from a 5µm diameter core single-mode fiber, illuminating the system from a distance of ∼1.5m. The incident beam was expanded by an engineered diffuser with 20° circular divergence, providing a near tflat intensity profile, followed by a 220 grit ground glass diffuser to act as the surface to be imaged. The double telecentric system was composed of two identical infinite conjugate achromatic doublet lenses of focal length f=75mm, placed 2f apart, with a variable aperture stop placed at the common focal point in the center. The ground glass to be imaged was fixed at the outer focal point of one lens, while the SPAD array was mounted at the outer focal point of the other on a six-axis translation stage to allow fine adjustment in the x,y, and z axes as well as roll, yaw, and pitch. This fine adjustment control for the SPAD array was used to ensure that the array was positioned perpendicular to, and centered on, the optical axis of the 4f system. This optical system was used to characterize two parameters. First, the uniformity was assessed in order to provide an indication of the performance consistency of the microlenses over the array. To do this we calculated the mean μ and the standard deviation σ of the counts recorded by each pixel. These are defined according to Eqs. (4) and (5), respectively, where $C_{i}$ is the counts recorded in pixel i, of total pixels n:

(4) $$\mu =\frac{1}{n}\sum_{i=1}^{n}C_{i},$$

(5) $$\sigma =\sqrt{\frac{1}{n}\sum_{i=1}^{n}{\left(C_{i}-\mu \right)}^{2}}.$$

The uniformity is then expressed as the coefficient of variation (CV), which is the ratio of standard deviation to mean, in Eq. (6), and it is presented as a percentage:

(6) $$CV=\frac{\sigma }{\mu }.$$

The second parameter considered was the concentration factor (CF), which provides a measure of the average enhancement of the effective fill-factor provided by the microlenses. Originally proposed in Ref. [43], this factor can be described by the total counts recorded by the microlens-integrated SPAD detector array divided by those recorded by the bare detector array under the same conditions (i.e., illumination power, wavelength, f-number, bias voltage, etc.), in Eq. (7):

(7) $$CF=\frac{\sum C_{lens}}{\sum C_{bare}}.$$

For each set of calculations, all measurements were conducted in complete darkness (except the illumination source) and at room temperature. A background measurement was taken of the system with the illumination path blocked so that light entering the detector without travelling through the optics can also be discounted. For both detector arrays, data was collected as a series of frames composed of the summation of a number of camera exposures using photon-counting mode. For the MiSPIA sensor, 16,384 exposures of 10µs each were collected per frame. Fifteen frames were collected to provide a total exposure time of 2.46 s. For the MF32 sensor, 40 exposures of 128µs duration were collected per frame. One thousand frames were used to provide a total exposure time of 5.12 s.

**Fig. 12.**
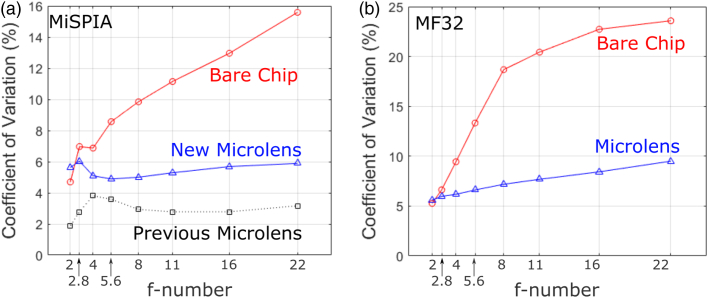
Comparison of uniformities according to the CV at the design wavelength as a function of illumination f-number using (a) MiSPIA at λ=808nm and (b) MF32 at λ=580nm. The lower value for CV represents a more uniform light distribution over the array.

## 5. RESULTS

To ensure the results were based solely on correctly functioning SPAD detectors, we removed all data from hot pixels before analysis. These pixels were identified according to the method described in Ref. [44], which applies a statistical cutoff to the dark count distributions of each detector. Hot pixels accounted for 31 pixels (3.0%) in the MiSPIA bare detector array, 34 pixels (3.3%) in the MiSPIA microlensed array, 215 pixels (21.0%) in the MF32 bare array, and 219 pixels (21.4%) in the MF32 microlensed array.

**Fig. 13.**
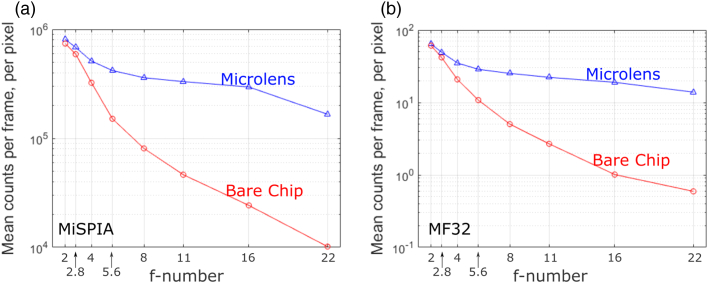
Mean counts recorded by the microlens chip (blue triangles) compared to the bare chip (red circles), at varying f-numbers, for (a) MiSPIA and (b) MF32. Average counts are recorded for the design wavelength in each case.

### A. Uniformity

To ensure the lens arrays are fabricated to a high quality, they should demonstrate a good homogeneity in performance. The CV was calculated for each, describing the uniformity of the illumination over the detector array, and is plotted by f-number at the lens design wavelengths λ=808nm and λ=580nm for the MiSPIA and MF32 SPAD detector arrays, respectively, in Fig. 12. It can be seen that the variation across the array is significantly more consistent at all f-numbers for the microlensed chip. For comparison, the data from the previous paper [15] are plotted alongside. The newer, faster lenses show comparable, though slightly higher variation than the lenses of longer focal length.

The observed deterioration in uniformity in the bare chip is likely a consequence of diminishing counts as illumination f-number is increased, resulting in the variation of detector efficiency and dark count rate (DCR) across the device, dominating the measurement of the CV, as opposed to the uniformity of the incident laser beam profile. This reduction in counts as illumination f-number increases can be seen in Fig. 13, comparing the counts recorded by the bare chip to that of the microlensed device for both chips.

### B. Concentration Factor

**Fig. 14.**
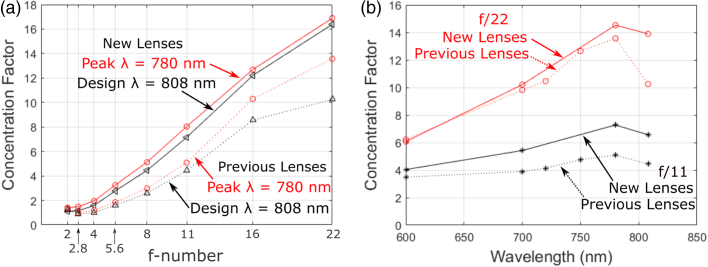
Concentration factor of MiSPIA microlens chip compared to the previous work [15]: (a) concentration factor at peak (red) and design (black) wavelengths as a function of illumination f-number for new (solid lines) and previous (dashed lines); and (b) concentration factor as a function of wavelength at f/22 (red) and f/11 (black) for new (solid lines) and previous (dashed lines) data.

**Fig. 15.**
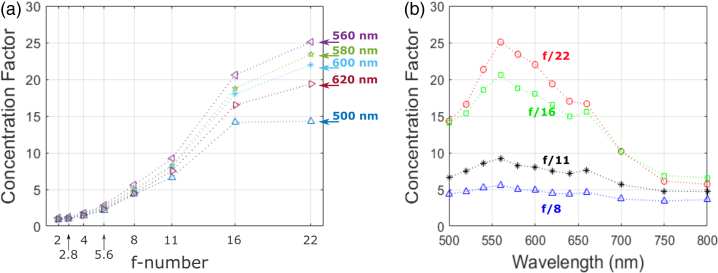
Concentration factor of MF32 microlens chip: (a) concentration factor at five wavelengths around the design wavelength (λ=580nm) as a function of illumination f-number; and (b) concentration factor as a function of wavelength at four illumination f-numbers.

The CF, as described by Eq. (7), provides a value representing the enhancement by use of microlenses to the detection capability of a detector array. The CF is the ratio of photon-detection events by the microlensed detector array to those of a bare detector array under the same conditions. To ensure a fair comparison, in each case the bare and microlensed SPAD detector arrays recorded the counts detected at each wavelength and for each illumination f-number, independently, while keeping the laser power level the same for all measurements. Additionally, background measurements were recorded for each measurement with the incident beam path through the optics blocked, which was then removed from the data. Measurements were made at eight illumination f-numbers: f/2,f/2.8,f/4,f/5.6, f/8, f/11, f/16, and f/22. For the MiSPIA SPAD detector array, data was collected for four wavelengths: λ=600, 700, 780, and 808 nm. To provide a more comprehensive trend, measurements at more wavelengths were recorded for the MF32 devices, collecting data at 12 wavelengths: λ=500, 520, 540, 560, 580, 600, 620, 640, 660, 700, 750, and 800 nm. Figures 14 and 15 illustrate the variation of concentration factors with (a) illumination f-number and (b) incident wavelength for the MiSPIA and MF32 arrays, respectively.

**Fig. 16.**
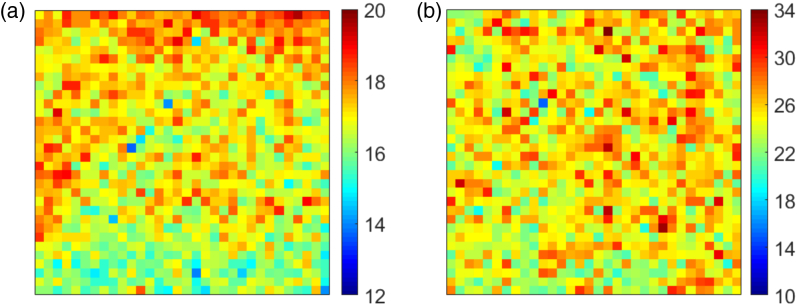
Spatial maps of concentration factor at f/22 on (a) MiSPIA at λ=780nm; (b) MF32 at λ=560nm.

From Fig. 14(a) it can be seen that the CF increases with illumination f-number as the maximum angle of incident light reduces. This is in line with expectation as the lenses were designed for a collimated incident source. From Fig. 14(b), the lens can be seen to perform most efficiently for the incident light at wavelength λ=780nm, with the CF reducing slightly for the design wavelength of λ=808nm at all f-numbers. The maximum achieved average CF was recorded as 16.9. Although designed specifically to focus collimated light at 808 nm wavelength, the concentration factor is greater than 1 at all wavelengths and all f-numbers measured. Furthermore, a greater CF is achieved at all f-numbers and wavelengths than that achieved previously, despite the use of a thinner substrate, while ensuring a low variation in performance across the DOE.

From Fig. 15(a) it is clear once more that the CF increases for all wavelengths with f-number and the reduction of maximum angle of incidence of incoming light. The wavelength of peak efficiency, Fig. 15(b), is found to be at λ=560nm. The maximum recorded average CF is 25.1, and once again values for CF of greater than 1 are obtained at all measured wavelengths and f-numbers. Figure 16 provides spatial maps showing the variation in CF across each array under f/22 illumination. For visual clarity, hot pixels, whose data had been removed, have been reconstructed using a 3×3 median filter. In this approach, the median value of the 8 pixels surrounding a discarded hot pixel is used to replace the missing data. These images not only show the relatively high uniformity of the lenses over the array, but we see the large number of pixels in each case achieving much higher than average concentration factors, with values as high as CF=19.5 and CF=33.8 for the MiSPIA and MF32 devices, respectively.

**Table 2.** Microlens Performance

		Focal Length	CF at f/22	Effective	Wavelength
Chip	Lens	in Substrate	Mean (Max)	Fill-factor	Peak (Design)
MiSPIA	Previous [15]	1000µm	13.6 (∼15.1^*a*^)	42.6%	780 (808) nm
MiSPIA	New	620µm	16.9 (19.5)	53.1%	780 (808) nm
MF32	New	190µm	25.1 (33.8)	38.7%	560 (580) nm

^*a*^ Estimated from [15].

The average CF of >25 represents a significant improvement on microlenses used with comparable sensors [12], while the uniformity across the DOE is considerably better than microlenses for which similar CFs have been achieved [45]. The key aspects of the performance of each microlens tested are highlighted in Table 2, showing the focal length, maximum recorded concentration factor, new effective fill-factor, and peak wavelength recorded compared to the design wavelength. In order to achieve a 100% fill-factor, the focusing efficiency would have to be perfect across the entirety of every pixel. However, there will be limitations caused by the finite number of phase levels and the finite bandwidth of incident light, as well as inevitable tolerances in the etching processes.

The maximum average CF for the microlensed MiSPIA chip represents an improvement on previous best CFs recorded for infinite conjugate microlenses integrated with MiSPIA chips of 13.6 [15]. Furthermore, this is achieved using a microlens array fabricated on a thinner substrate, requiring a faster microlens (shorter focal distance), using a substrate of 620µm thickness compared to the 1 mm thickness used in our previous work [15]. The exemplary quality of the microlens arrays fabricated are highlighted by the high degree of uniformity maintained across the array, with CV: 5%–10% under all illumination f-numbers, which can be hard to achieve with refractive elements [46]. While the peak wavelength for CF is close to the design wavelength, a slight shift is observed in both the MiSPIA and MF32 arrays. This can likely be attributed to tolerance errors in the fabrication process, while the additional thickness of the adhesive used to bond the microlens array to the chip (expected to be <10µm), which has not been taken into account during the design process, could also contribute to this offset. The concentration factors for the MF32 detector array are seen to be significantly higher than those recorded for the MiSPIA array, achieving a maximum CF of 33.8. This is largely due to the much smaller bare fill-factor—1.54% for the MF32 compared to MiSPIA’s 3.14%—therefore, allowing for a much greater gain. However, the MiSPIA detector array has a much larger maximum effective fill-factor of 53.1% compared to MF32’s 38.7 %. The smaller active area of the MF32 array (7µm diameter) presents a challenge of ensuring the focused spot is within this region, which we can relate to the linear tolerance of the alignment process—i.e., the margin for error in alignment. Assuming a perfect diffraction-limited spot size of 3.7µm, the margin is only 1.65µm. This linear tolerance is in comparison to the MiSPIA chip’s 12.2µm and explains the lower maximum total effective fill-factor achieved. Several factors are also likely to contribute to increasing the spot sizes to above the diffraction limit, reducing this linear tolerance further. First, the much smaller pixel dimensions require feature sizes approaching the fabrication limit, meaning a larger chance of fabrication errors in these areas. Second, the much faster lens means the depth of field of the focused spot is much shorter, and so the thickness of the adhesive will have a proportionally higher defocusing effect. Finally, the thinness of the substrate means that any deviation from the specified thickness across the substrate (i.e., inhomogeneity of the substrate thickness), or deformation of the substrate, will also have a proportionally larger defocusing effect. Losses will also occur due to Fresnel reflection at each interface, while there also exists the potential for thin film interference effects at lens, optical adhesive, and SPAD interfaces, which may reduce transmission further.

## 6. CONCLUSIONS

High concentration factor diffractive microlens arrays with low coefficients of variation have been fabricated using binary-mask-based photolithography and reactive ion etching. These have been integrated with two different 32×32 Si CMOS SPAD image sensors. Without microlenses, the MiSPIA and MF32 sensors have fill-factors of 3.14% and 1.54%, respectively. Analysis was conducted over a large spectral range (λ=500−900nm) and illumination f-number range (f/2−f/22), allowing a full characterization of each lens array under different conditions using CF and CV as parameters of interest. The vastly reduced CV at all except the lowest illumination f-number (f/2) in both instances demonstrates the high-quality, uniform performance achievable with diffractive microlenses fabricated in this manner. The largest CF achieved with the MiSPIA detector array is 19.5, while the maximum CF achieved with the MF32 array is 33.8. This represents a dramatic improvement in concentration achievable over a highly uniform array and demonstrates the significant benefits, in terms of light collection, which can be expected through the use of microfabricated diffractive lenses integrated with image sensors of low fill-factor. The benefits are particularly useful in highly photon-starved environments such as those experienced in applications in biomedicine, for example, fluorescence lifetime imaging [8] or positron emission tomography [47], time-of-flight ranging, and LIDAR [48]. These types of lenses are likely to be of most benefit for use with CMOS-incompatible arrays [30,31] and novel device architectures such as Ge-on-Si SPADs [32,33], that operate in the short-wavelength infrared (SWIR) band. The ability of diffractive optics to operate as high-efficiency microconcentrators, without otherwise compromising performance across large-format arrays, makes this form of microlens a versatile and very high performance option for enhancement of low fill-factor detector arrays.
